# Minithoracotomy oesophagectomy for oesophageal carcinoma with aberrant right subclavian artery: a rare case of dysphagia

**DOI:** 10.1186/1471-230X-14-163

**Published:** 2014-09-21

**Authors:** Duminda Subasinghe, Hemantha Sudasinghe, Chathuranga Tisara Keppetiyagama, Sumana D Handagala, Anuja Abayadeera, Merrenna IM De Zoysa

**Affiliations:** University Surgical Unit, The National Hospital of Sri Lanka, Colombo, Sri Lanka; Department of Thoracic Surgery, National Hospital for Respiratory Diseases, Welisara, Sri Lanka

**Keywords:** Aberrant subclavian artery, Oesophageal carcinoma, Dysphagia lusoria

## Abstract

**Background:**

Aberrant R/subclavian artery is a rare congenital anomaly involving aortic arch. Oesophageal carcinoma with associated aberrant R/subclavian artery is very rare and only few cases has been reported in literature. If unrecognized and injured during oesophageal surgery, it can lead to disastrous complications. When associated with oesophageal carcinoma, it can cause diagnostic confusion as the symptoms are similar.

**Case presentation:**

A 60 year old previously healthy female presented with intermittent dysphagia, odynophagia and loss of weight of 3 months duration. She was found to have a oeophageal carcinoma with incidentally co-existing aberrant R/subclavian artery.

**Conclusion:**

Although rare this entity should be considered as a differential diagnosis in a patient with dysphagia. In addition, pre-operative identification is important to prevent intra operative vascular complications. The diagnosis and treatment of this rare condition is discussed in this article.

## Background

An aberrant right subclavian artery (ARSCA) is an unusual congenital anomaly although a well described entity. ARSCA is often an incidental finding on imaging studies and it affects about 0.5% to 1.8% in the general population [1]. In over 80% of cases, the location is posterior to the oesophagus [2]. When associated with oesophageal carcinoma, it can cause diagnostic confusion as the symptoms are similar.

In normal individuals the right subclavian artery (RSCA) arises from the brachiocephalic artery. Embryologically, the proximal part of the RSCA develops from the right fourth aortic arch and the distally from the seventh intersegmental artery [3]. In ARSCA, abnormal development results from degeneration of the entire right fourth arch. The right seventh intersegment artery persists in its attachment to the distal descending aorta [4]. The ARSCA consequently originates from the aorta distal to the left subclavian artery, and passes behind the oesophagus and trachea toward the right [4]. The right recurrent nerve is also abnormal and passes directly to the larynx from the vagus rather than “recurring” from the chest [4].

If unrecognized and injured during open or minimally invasive oesophageal surgery, it can lead to disastrous complications. Therefore as a surgeon, awareness about this anomaly is important in order to prevent catastrophic hemorrhage from laceration of the ARSCA. Transhiatal oesophagectomy for cancer is proposed in order to decrease postoperative morbidity [5]. Mediastinal haemorrhage during transhiatal oesophagectomy occurs in 1% to 9% of procedures, usually from injury to the aorta or azygous vein [6, 7].

## Case presentation

**Figure 1:**
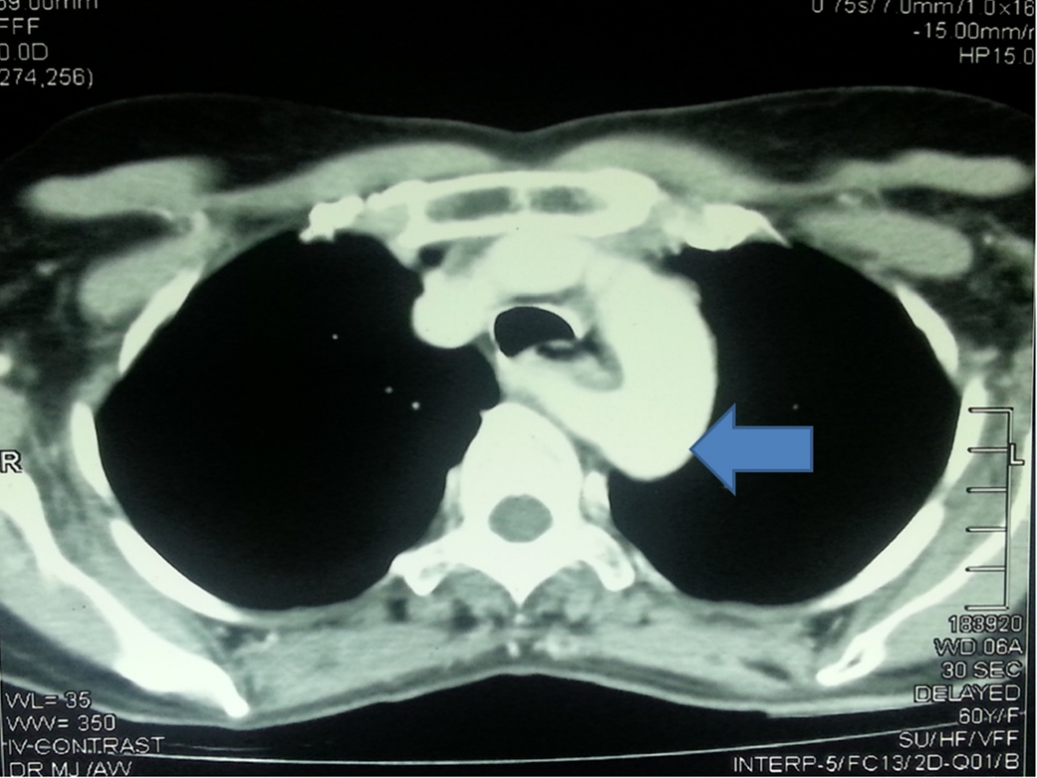
**Contrast enhaced CT thorax showing the course of aberrant R/subcalvian artery behind the oesophagus (blue arrow shows the aberrant vessel).**

A 60 year old previously healthy female presented with intermittent dysphagia, odynophagia and loss of weight of 3 months duration. Her symptoms were non progressive. She was otherwise well. Physical examination was unremarkable except her BMI was 16 kg/m^2^. Her upper gastrointestinal endoscopy revealed a superficial mucosal irregularity from 25–35 cm with friability and contact bleeding. There was no evidence of external compression of the oesophagus on endoscopy. The biopsy of oesophageal lesion revealed a well differentiated squarmous cell carcinoma. Her preoperative contrast enhanced CT of thorax and abdomen showed a normal oesophagus without any luminal lesions or wall thickening. However, it showed an abnormal right subclavian artery (Figure 1) running behind the thoracic oesophagus above the carina. There was no evidence of locoregional spread, mediastinal lympadenopathy or evidence of distant metastases. Therefore she was planned for a transthoracic oesophagectomy.

**Figure 2:**
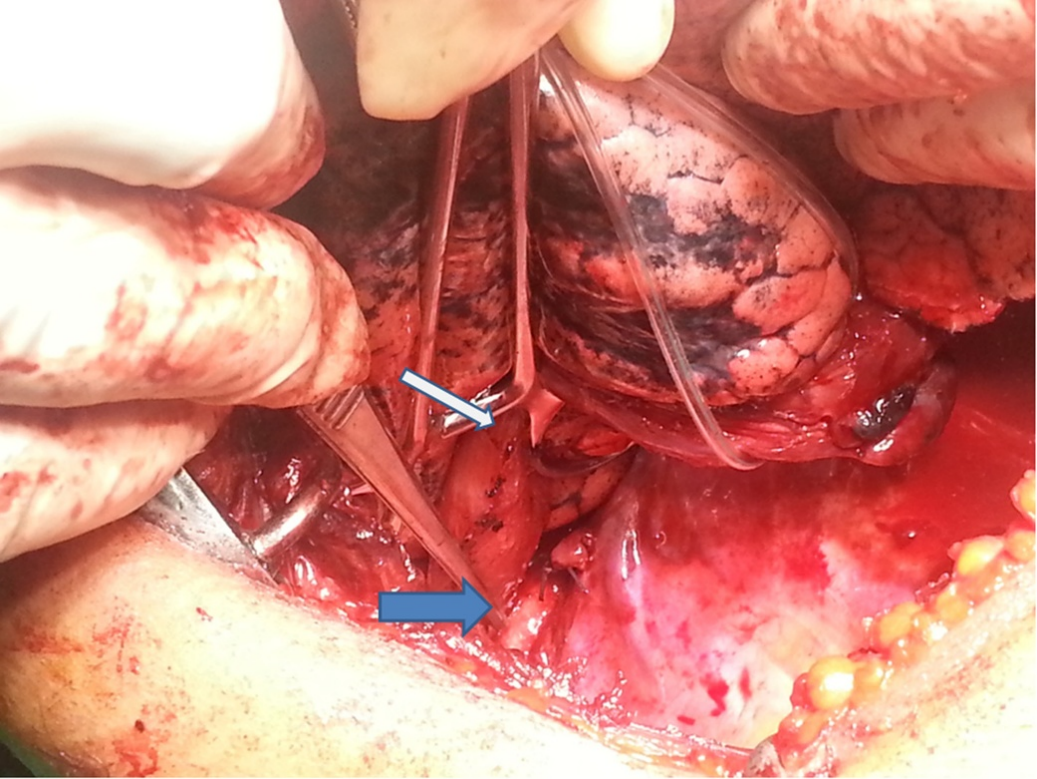
**Aberrant R/subclavian artery (blue arrow) identified following mobilization of thoracic oesophagus (white arrow).**

**Figure 3:**
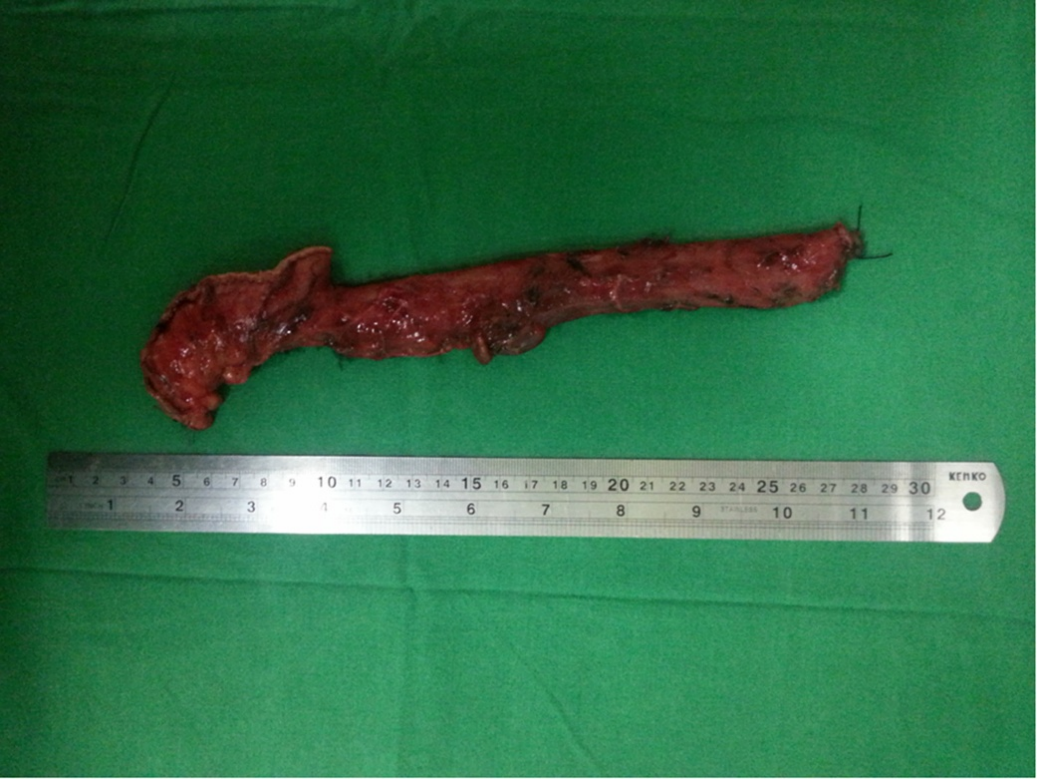
**Resected specimen of oesophagus.**

The thoracic cavity was approached by a mini thoracotomy [8] as it provides adequate access to the oesophagus, aberrant vessel and associated with less post-operative respiratory complications when compared to conventional posterolateral thoracotomy. The oesophagus was mobilized from lower end to up wards. During thoracic oesophageal mobilization, there was an abnormal retro-oesophageal R/subclavian artery arising from aortic arch above the carina (Figure 2). An upper abdominal incision followed by a cervical incision were also made to mobilize the stomach and create a cervical oesophago-gastric anastomosis. Mediastinal lymphadenectomy was done. Cervical lymphadenectomy was not performed. The patient was managed in the surgical intensive care unit for 6 days. On post-operative day 2, she was extubated and gradually mobilized and started on chest physiotherapy. During this period she was well and recovery was uneventful. She was transferred to the surgical ward on post operative day seven and died suddenly most probably due to cardiac arrest or massive pulmonary embolism. Histopathological analysis of resected specimen of oesophagus (Figure 3) showed a well differentiated squarmous cell carcinoma (pT1N0R0).

## Discussion

Dysphagia is a common problem encountered in surgical practice and result in low quality of life in patients. Oesophageal dysphagia could be caused by oesophageal carcinoma, benign stricture and webs, achalasia, diffuse oesophageal spasm and scleroderma [9]. Although, ARSCA (lusoria artery) is a rare cause of dysphagia it’s the most common abnormality of the aortic arch. Dysphagia lusoria was first described by Bayford in 1787 [10]. Intraoperative damage to this artery during oesophageal surgery may lead to disastrous complications. Only few cases of oesophageal cancer with associated ARSCA are reported in literature [11–14].

The surgical approach to oesophageal carcinoma is determined by the location of the tumour as well as the surgical expertise available. Transhiatal oesophagectomy (THE) for cancer is proposed in order to reduce the postoperative morbidity. The major inconvenience during the THE is blind mediastinal dissection. In addition, the presence of ARSCA can complicate the preoperative or postoperative course especially due to fistula formation and bleeding [11, 15]. Therefore we planned for a transthoracic procedure in our patient. Mediastinal hemorrhage during THE occurs in 1% to 9% of procedures, usually from injury to the aorta or azygous vein [7]. An ARSCA is also at significant risk for injury during mobilization of the oesophagus through the cervical and transhiatal routes. Pre-operative diagnosis will require a very high index of suspicion and radiologic investigations. This was evident in our case because oesophagus was patent on upper gastrointestinal endoscopy except an area of mucosal irregularity which could not explain the degree of dysphasia in our patient. This also confirmed on contrast CT thorax and abdomen where the oesophageal was normal thickness without any evidence of growth. But it showed an abnormal retro-oesophageal artery.

## Conclusion

Therefore we would conclude that although rare this entity should be considered as a differential diagnosis in a patient with dysphagia. In addition, pre-operative identification and careful dissection during oesophageal mobilization will help in preventing disastrous vascular complications in patients with an ARSCA who undergo oesophagectomy.

## Consent

Written informed consent was obtained from the patient and relatives for publication of this case report and any accompanying images. A copy of the written consent is available for review by the Editor of this journal.
